# CMC-Enabled PEDOT:PSS Film for High-Performance Electrochromic Material

**DOI:** 10.3390/polym18020263

**Published:** 2026-01-19

**Authors:** Ruiying Zhang, Yuanyuan Liu, Chaoqun Ji, Fengwei Sun, Shan Lin, Xu Cao, Qiang Wang, Lihui Chen, Jianguo Li

**Affiliations:** 1 State Key Laboratory of Biobased Material and Green Papermaking, Qilu University of Technology, Shandong Academy of Sciences, Jinan 250353, China; 2 National Forestry and Grassland Administration Key Laboratory of Plant Fiber Functional Materials, College of Material Engineering, Fujian Agriculture and Forestry University, Fuzhou 350002, China; 3 Fujian Laile Biotechnology Co., Ltd., Fuzhou 350002, China

**Keywords:** cellulose derivative, electrochromic, stability, coloring efficiency

## Abstract

Flexible, intelligent color-changing windows with excellent color-switching capability and fast response time rely significantly on conductive composite layers composed of electrochromic materials and flexible, transparent substrates. Herein, we developed a high-performance electrochromic film (CPC) with mechanical, humidity, and temperature insensitivity by coating sodium carboxymethyl cellulose (CMC)-dispersed PEDOT:PSS onto a CA film. In the CPC system, CMC not only effectively enhances interfacial bonding and compatibility between the hydrophobic CA film and the hydrophilic PEDOT:PSS but also enables uniform and stable deposition of PEDOT:PSS on the CA film. As a result, the designed CPC demonstrates a high optical modulation capability (the transmittance changes from 60.1% to 3%) and a response time of 2 s. In addition, the CPC features the advantages of mechanical-, humidity-, and temperature-insensitive electrochromic distinctions, where it outputs the stable coloring efficiency with 100-time bending treatment, various temperatures, and humidity in an all-day outdoor environment. The developed CPC electrochromic film offers new insights into promoting the structural simplification and sustainability of electrochromic materials.

## 1. Introduction

Flexible, intelligent color-changing windows represent highly innovative applications that utilize advanced materials and technologies to achieve new functionalities and enhance the user experience [1,2,3]. Electrochromic devices (ECDs) have attracted significant attention owing to their high coloration efficiency, wide optical modulation range, and quick switching speed under low electric fields, making them promising candidates for intelligent color-changing windows [4,5,6]. Typically, ECDs are composed of a transparent substrate, conductive materials, and an electrolyte [7,8]. Poly(3,4-ethylenedioxythiophene):poly(styrenesulfonate) (PEDOT:PSS) is commonly used as an organic electrochromic and conductive material with excellent color-switching ability, fast response time, and good electrical conductivity [9,10]. For instance, Eren et al. (2019) [11] designed a PEDOT/poly(3-methylthiophene)-based ECD with a conductivity of 132.3 Ω sq^−1^, an optical modulation range of 32.2% and a response time of ~0.4 s. Janthajam et al. (2025) [12] fabricated PEDOT:PSS/gold nanoparticle ECDs with an optical modulation range of 20% and a response time of 48 s.

In addition, a transparent substrate, such as glass or a synthetic polymer, plays a key role in constructing high-performance ECDs. However, the polymer and glass materials are commonly hydrophobic, making it difficult to uniformly deposit the hydrophilic PEDOT:PSS on these substrates, thereby compromising optical modulation stability. Therefore, existing strategies predominantly involve integrating PEDOT:PSS with other components to construct ECDs [13,14,15,16]. For example, Li et al. (2021) [17] printed conductive ink of PEDOT:PSS on an indium tin oxide-coated polyethylene terephthalate (ITO-PET) flexible substrate, followed by depositing W_18_O_49_ on the PEDOT: PSS/ITO-PET to fabricate a sandwich-structured ECD with a transmittance modulation of 63.3% (from 80% to 16.7%), and response times of ~20 s. Lv et al. (2023) [18] also assembled a similar structured ECD, including a first layer made of silver nanowire, a second layer of PEDOT:PSS, and third layer of poly(3,4-propylenedioxythiophene) on a PET substrate to obtain laterally configured ECDs with transmittance modulation of 59.5% and a response time of ~5 s. Despite these advances, the fabrication of layer-structured composite ECDs commonly features complex operations, high costs and weak reproducibility/stability due to the weak interface compatibility in the multiple layers.

**Figure 1:**
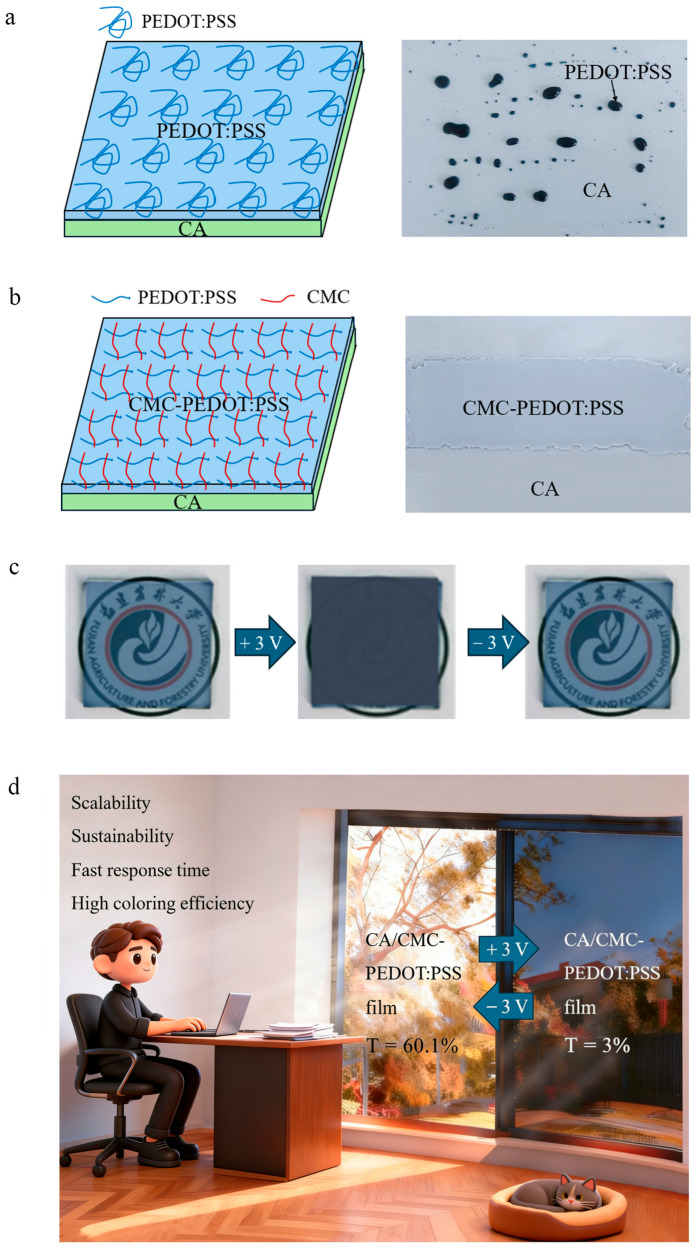
(**a**) The structure of the CA/PEDOT:PSS film. (**b**) The structure of the CPC film. (**c**) Optical modulation capability of the CPC film when a voltage is applied. (**d**) Intelligent color-changing windows based on the CPC. T, transmittance.

To address these problems, we introduced CMC, a film-forming agent of cellulose derivatives, to strengthen the interface bonding and compatibility between hydrophobic CA and hydrophilic PEDOT:PSS (Figure 1a,b) [19,20,21,22]. This modification effectively resolves the interfacial compatibility issue between CA and PEDOT:PSS, thereby improving the mechanical stability and electrochemical performance of the composite film while also simplifying the fabrication process compared to layer-structured approaches. Moreover, employing cellulose and its derivatives not only fulfills key functional roles but also enhances the economic viability of ECDs due to their low cost and natural abundance.

Herein, we prepared a high-performance, mechanical-, humidity-, and temperature-insensitive electrochromic film by coating sodium carboxymethyl cellulose (CMC)-dispersed PEDOT:PSS onto a CA film to prepare a CPC (CA/PEDOT:PSS-CMC) electrochromic film. The electrochromic film exhibits excellent performance with high transmittance (60.1%), low resistance (76.3 Ω·sq^−1^), and extremely high optical modulation capability (ΔT = 57.1%) within 2 s. The color change of the CPC films upon voltage application is shown in Figure 1c. Moreover, they demonstrate excellent stability after 80-time color–decoloring cycle tests, 100-time bending cycles, or 24 h outdoor tests. The exceptional properties of the film make it extremely promising for use in intelligent color-changing windows, as illustrated in Figure 1d.

## 2. Experiments

### 2.1. Materials

Bamboo dissolving pulp fiber was purchased from Fujian Qingshan Paper Co., Ltd., Sanming, China. Dimethyl Sulfoxide (DMSO) and sodium carboxymethyl cellulose (CMC) were acquired from Shanghai Aladdin Biochemical Technology Co., Ltd., Shanghai, China. Poly 3,4-ethylenedioxythiophene/polystyrene sulfonate (PEDOT:PSS) was bought from Xi’an Paulette Optoelectronics Technology Co., Ltd., Xian, China.

### 2.2. Preparation of CPC (CA/PEDOT:PSS-CMC) Electrochromic Film

First, 0.526 mL DMSO was added to 10 mL PEDOT:PSS solution. Then, CMC solutions of varying concentrations were added to sonicated PEDOT:PSS solutions of different concentrations to prepare CMC-PEDOT:PSS with various ratios. Meanwhile, the cellulose acetate (CA) film was cut into 3 cm × 3 cm specimens, and the prepared CMC-PEDOT:PSS solution was poured onto the CA film and coated to obtain CPC electrochromic films.

### 2.3. Characterization

The surface morphologies of the samples were evaluated using a field-emission scanning electron microscope (FESEM; SU8010, Hitachi, Tokyo, Japan) at an acceleration voltage of 20 kV. Atomic force microscopy (AFM; Bruker Multimode 8, Bruker, Karlsruhe, Germany) was employed to characterize the sample morphology in detail and to measure surface roughness. The optical properties of the samples were evaluated using an ultraviolet–visible spectrophotometer (UV-1800, SHIMADZU, Kyoto, Japan). The resistance of the sample was measured via a four-point probe (KDT-5, Guangzhou Kunde Technology Co., Ltd., Guangzhou, China). For the electrochromic performance test, the CA electrochromic film (as the positive electrode) and indium tin oxide (ITO, as the negative electrode) were connected to a constant voltage. Subsequently, the electrode polarity was reversed, and the test was repeated using the same method. For the bending cycle test, the samples were bent via a single-column table model (34SC-1, Instron, Norwood, MA, USA) with a cycling rate of approximately 1 cycle/s.

## 3. Results and Discussion

### 3.1. The Effect of CMC on the CPC Electrochromic Film

As we know, PEDOT:PSS is coated on a transparent substrate (such as cellulose derivatives), commonly as a water solution, to construct electrochromic devices. Due to the high hydrophobicity of CA, a kind of cellulose derivative with high transmittance and flexibility, it is hard to uniformly deposit the water-soluble PEDOT:PSS and form a thin layer on the hydrophobic surface of CA film, as displayed in Figure S1. In order to strengthen the interface bonding and compatibility between hydrophobic CA and hydrophilic PEDOT:PSS, CMC, a film-forming agent made of cellulose derivatives, is further introduced. Based on this consideration, the CMC makes the PEDOT:PSS solution viscous and allows a uniform thin layer of PEDOT:PSS to form on the CA film. In addition, due to the similar molecular structures of CMC and CA (both are cellulose derivatives), the CMC also improves the interfacial strength and adhesion capability between CA and PEDOT:PSS.

**Figure 2:**
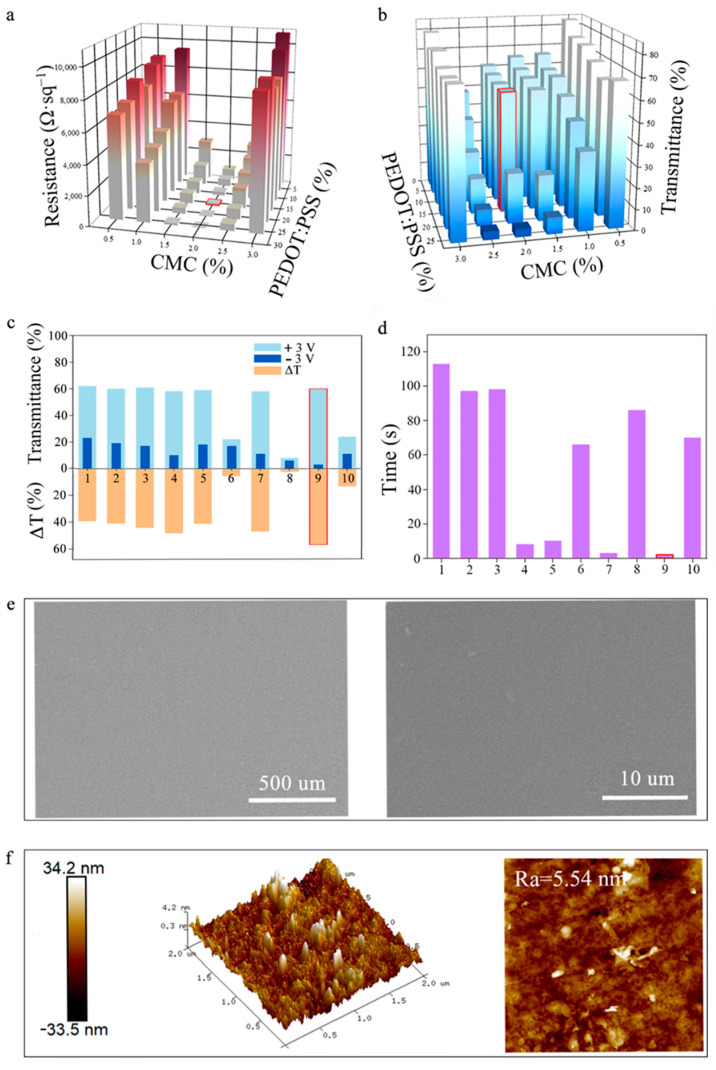
(**a**) Effect of CMC and PEDOT:PSS concentrations on the conductivity of CPC film. (**b**) Effect of CMC and PEDOT:PSS concentrations on the transmittance of CPC film. (**c**) Optical modulation capability of CPC film upon application of a 3 V voltage. (**d**) Response time of CPC film. The 9th sample shows optimized electrochromic performance, with the large optical modulation capability (ΔT = 57.1%) and a short response time (2 s). (**e**) SEM images of the 9th CPC film. (**f**) AFM images of the 9th CPC film with surface roughness (Ra) of 5.54 nm.

We first prepared 36 solutions with varying concentrations of PEDOT:PSS and CMC. When the concentration of CMC is too low, the bonding capability of CMC is too weak to enable the deposition of the PEDOT:PSS onto the CA film, resulting in high resistance and high transmittance (less PEDOT:PSS deposition), as depicted in Figure 2a,b. Conversely, an excessively high concentration of CMC leads to an overly viscous state, causing difficulty in coating the PEDOT:PSS on the CA film, which also leads to less PEDOT:PSS on the developed CPC electrochromic film, as displayed in Figure S2. Among those tested, 10 solutions successfully formed electrochromic films, as detailed in Table 1.

As displayed in Figure 2c, the sixth and eighth samples have a weak optical modulation capability with a low transmittance change degree (ΔT of 6% and 3%, respectively), due to the high amount of PEDOT:PSS (high concentration in the CMC/PEDOT:PSS solution). Photos of coatings for the third, sixth, seventh, and ninth samples (red frame)are exhibited in Figure S3. Among the prepared CMC/PEDOT:PSS solutions, the ninth sample shows the highest optical modulation capability, which is 57.1% from 3% to 60.1%. In addition, the ninth CPC electrochromic film has the lowest conductive resistance, 76.3 Ω·sq^−1^, which supports low-voltage operation during electrochromic operation. We also investigated the voltage-response capability, and the response time was 2 s for the ninth sample (Figure 2d). As a result, by optimizing the CMC and PEDOT:PSS concentrations, we can primarily prepare the CMC/PEDOT:PSS solution and thus construct a superior CPC electrochromic film.

As shown in Figure 2e, the microstructural analysis reveals a smooth, uniform surface on the CPC film (ninth sample). More detailed morphological features are observed in Figure 2f, where the developed CPC film features a low surface roughness of 5.54 nm. All the above results demonstrate that the CMC enables a uniform, smooth PEDOT:PSS layer on the CA film.

### 3.2. Electrochromic Characterization

**Figure 3:**
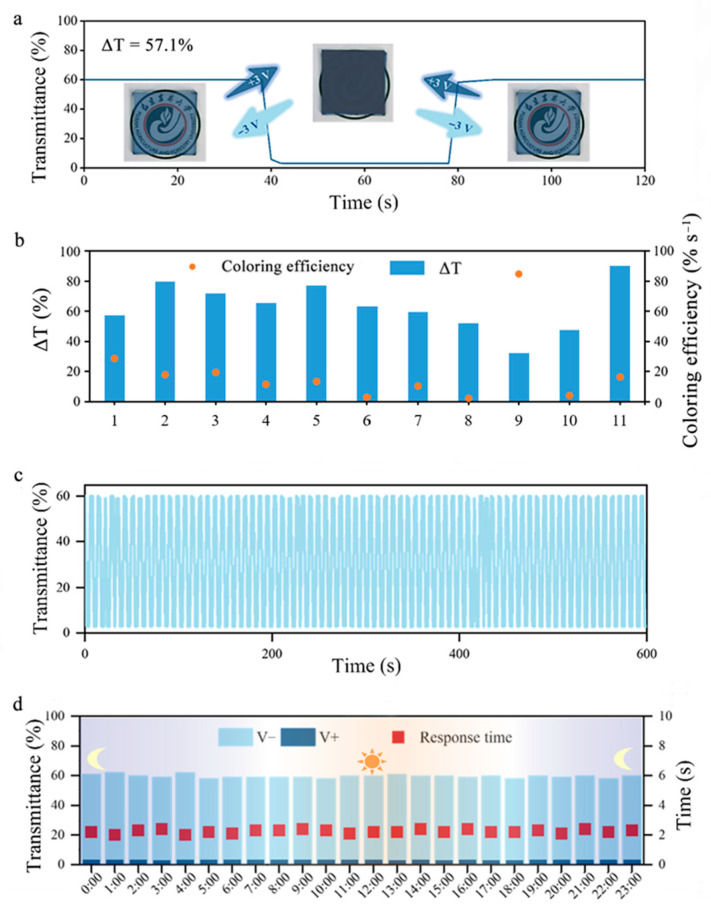
(**a**) Optical modulation capability of the CPC film upon application of a 3 V voltage. (**b**) Comparison of the optical modulation capability (ΔT) and coloring efficiency of various electrochromic materials. 1, CPC; 2, W_17_O_47_-(NaWO_3_-knots)/PEDOT:PSS; 3, WO_3_/PEDOT; 4, Ag nanowires/PEDOT:PSS/WO_3_ nanoparticles; 5, glass–(WO_3_/PEDOT:PSS); 6, glass–PET (W_18_O_49_/PEDOT:PSS); 7, PET-(PEDOT:PSS-AgNWs); 8, PET-PEDOT:PSS; 9, PC-(PEDOT/Ch:PEDOT:PSS:LiTRIF/PMeT); 10, SEBS-(PEG/Ag/PDMS/PEDOT:PSS); 11, PDMS-(Cu/PEDOT:PSS/Xylitol/graphene). (**c**) Optical modulation capability of the CPC film under 80-time color–decoloring cycling. (**d**) Optical modulation capability of the CPC film under 24 h outdoor test.

To further evaluate the electrochromic performance of the CPC electrochromic film, transmittance measurements are conducted at an applied voltage of 3 V. As shown in Figure 3a, the CPC film exhibits a high transmittance of 60.1% in the absence of an applied voltage. When a forward voltage is applied (inducing PEDOT:PSS oxidation), the transmittance of the CPC film decreases from 60.1% to 3%. As a result, the CPC film demonstrates a high optical modulation capability with a ΔT of 57.1%. In addition, due to high conductivity (76.3 Ω·sq^−1^ in Figure 2a), the response time and applied voltage following the electrochromic function of CPC film become very low, and they are 2 s and 3 V. Herein, we also investigated the coloring efficiency using the following equation: Coloring efficiency = ΔT (Optical modulation capability)/Average response time = $\frac{T_{1}-T_{2}}{(t_{1}+t_{2})/2}$. The CPC film exhibits the high coloring efficiency, about 28.6% s^−1^ as shown in Figure 3b and Table 2, which is much superior to that of most previously reported electrochromic materials [11,17,18,23,24,25,26,27,28,29].

Notably, the CPC film also exhibits exceptional durability. As shown in Figure 3c and Figure S4a, it maintains a stable optical modulation capability over 80-time color–decoloring cycle tests, with transmittance consistently varying between 3% and 60.1%. Furthermore, its working stability is tested under all-day outdoor conditions. Although fluctuations of temperature and humidity take place (Table S1), the CPC film maintains stable optical modulation capabilities and coloring efficiency throughout the rest of the day (Figure 3d and Figure S4b). Overall, the CPC electrochromic film demonstrates significant advantages in optical modulation and response time, coupled with excellent stability.

### 3.3. Stability and Application

In practical applications, ECDs may undergo various deformations when enduring external forces, so stability under bending conditions is particularly important. We also investigated the optical modulation capabilities when bending the CPC electrochromic film. As shown in Figure S5a and Figure S6, despite the 100-time bending treatment with a bending radius of 1 cm, the CPC film features a smooth surface, and correspondingly, a relatively high conductivity with a low resistance of ~76.3 Ω·sq^−1^. As a result, our CPC film demonstrates a superior optical modulation capability of 57.1% and a response time of 2 s, with various bending radii from 0.1 mm to 10 mm (Figure S5b).

**Figure 4:**
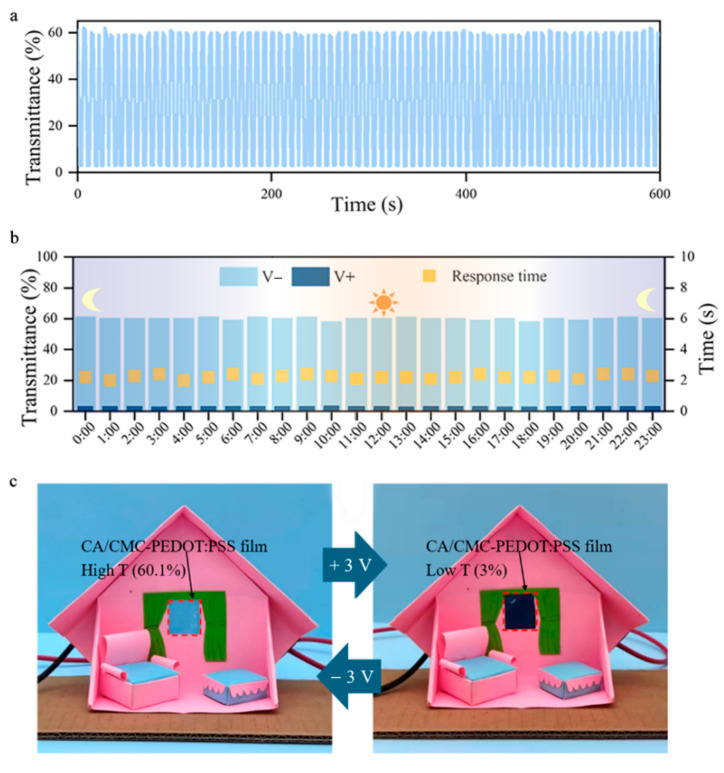
(**a**) Optical modulation capability of the 100-time-bent CPC film with high coloring–decoloring stability. (**b**) Optical modulation capability of 100-time-bent CPC film under a 24 h outdoor test. (**c**) Photos of the application of the CPC film in intelligent color-changing windows.

Additionally, the CPC film with 100-time bending treatment still enables a low transmittance of 3% at a forward operating voltage of 3 V, and high transmittance of 60.1% at a reverse operating voltage of 3 V. Interestingly, the optical modulation capability is still stable after 80-time color–decoloring cycle tests (Figure 4a and Figure S7a). As shown in Figure 4b and Figure S7b, in the outdoor environment with all-day testing, the CPC film demonstrates the desirable electrochromic distinction, and the optical modulation capability and response time are not affected by the varying temperature and humidity.

The CPC electrochromic film boasts unique optical modulation capabilities, excellent electrical conductivity, and low production costs, thus facilitating its widespread application across numerous technical fields, such as intelligent color-changing windows. A simulation of its application in an intelligent color-changing window is presented in Figure 4c. Herein, the CPC electrochromic film enables adjustable transmittance. During the daytime with abundant sunlight, the film switches to a dark blue color (transmittance of 3% at 3 V), effectively blocking the solar radiation, thereby contributing to energy conservation and sun protection. At night or in low-light conditions, it maintains high transmittance (60.1%) and visibility, ensuring a clear view. Overall, these properties of the CPC electrochromic film not only enhance its feasibility for practical applications in diverse scenarios but also provide new opportunities for the design of more complex flexible electronic devices.

## 4. Conclusions

In this study, we prepared a stable CPC electrochromic film by introducing CMC, a cellulose-derived crosslinking agent, to enhance interfacial bonding and compatibility between the hydrophobic CA and hydrophilic PEDOT:PSS. This CPC electrochromic film exhibits excellent performance, including a high transmittance of 60.1%, a low resistance of 76.3 Ω·sq^−1^, and a relatively high optical modulation capability of 57.1% within 2 s (coloring efficiency of 28.6% s^−1^). In addition, it also demonstrates outstanding working stability of optical modulation capability, even enduring the 80-time color–decoloring cycling, 100-time bending treatment, and 24 h outdoor environment. The electrochromic performance of the CPC film outperforms that of most reported materials, paving the way to enable the advancement of ECDs.

## Figures and Tables

**Table 1:** The solutions with different CMC-to-PEDOT:PSS ratios.

Samples	CMC Concentration (%)	PEDOT:PSS Concentration (%)
1	1.5	5
2	1.5	10
3	2	1
4	1.5	15
5	2	5
6	1.5	20
7	2	10
8	1.5	25
9	2	15
10	2	20

**Table 2:** Comparison of the performance of various electrochromic materials.

Electrochromic Materials	Resistance (Ω·sq^−1^)	Response Time (s)	Optical Modulation Capability (%)	Coloring Efficiency (% s^−1^)	Voltage (V)	Sustainability	Reference
1. CPC	76.3	2	57.1	28.6	±3	High	This work
2. W_17_O_47_-(NaWO_3_-knots)/PEDOT:PSS	-	3.5–5.5	79.7	17.7	±1	Middle	[23]
3. WO_3_/PEDOT	-	3.6–3.8	72	19.5	±1	Middle	[24]
4. Ag nanowires/PEDOT:PSS/WO_3_ nanoparticles	60–85 Ω/10 cm	2.5–9	65.5	11.4	−0.7~0.1	Middle	[25]
5. Glass-(WO_3_/PEDOT:PSS)	-	5.7–6	77	13.2	−1.2~0	Middle	[26]
6. Glass-PET (W_18_O_49_/PEDOT:PSS)	-	18.7–23.2	63.3	3	±1.5	Low	[17]
7. PET-(PEDOT:PSS-AgNWs)	10.7	3.9–7.4	59.5	10.5	±0.9	Low	[18]
8. PET-PEDOT:PSS	-	17.6–28.5	52	2.3	−1.8~0.4	Low	[27]
9. PC-(PEDOT/Ch:PEDOT:PSS:LiTRIF/PMeT)	132.3	0.24–0.52	32.2	84.7	±2	Low	[11]
10. SEBS-(PEG/Ag/PDMS/PEDOT:PSS)	39–87	3.3–20.1	47.7	4.1	−1.2~0	Low	[28]
11. PDMS-(Cu/PEDOT:PSS/ Xylitol/graphene)	81–97	5.76–5.34	90	16.2	+14.9~+15.1	Low	[29]

## Data Availability

The original contributions presented in the study are included in the article/Supplementary Material, further inquiries can be directed to the corresponding authors.

## Supplementary Materials

The following supporting information can be downloaded at https://www.mdpi.com/article/10.3390/polym18020263/s1: Figure S1: Photos of (a) wet PEDOT:PSS on CA film and (b) dry CA/PEDOT:PSS film; Figure S2: Effect of CMC concentrations on the viscosity of CMC/PEDOT:PSS solution; Figure S3: Photo of CPC with various-time coatings: (a) 3rd, (b) 6th, (c) 7th, (d) 9th sample; Figure S4: Optical modulation capability of the CPC film (a) under 80-time color–decoloring cycle tests; (b) under 24 h outdoor test; Figure S5: (a) Resistance changes of CPC film after bending cycle tests under a 10 mm bending radius. (b) Optical modulation capability and response time of the CPC film after 100-time bending cycle tests under various bending radii; Figure S6: SEM images of the CPC film after 100-time bending cycles under a 10 mm bending radius; Figure S7: Optical modulation capability of the CPC film after 100-time bending cycles (a) under 80-time color–decoloring cycle tests; (b) under 24 h outdoor test; Table S1: Variations in temperature and humidity during the 24 h outdoor test.
