# Supplementary material for: CMC-Enabled PEDOT:PSS Film for High-Performance Electrochromic Material
Source: Polymers (Basel). 2026 Jan 19;18(2):263. doi: 10.3390/polym18020263 (PMC12845538; doi:10.3390/polym18020263)
Supplement: Supplementary file 1 [file polymers-18-00263-s001.zip › polymers-4091287-supplementary.pdf]

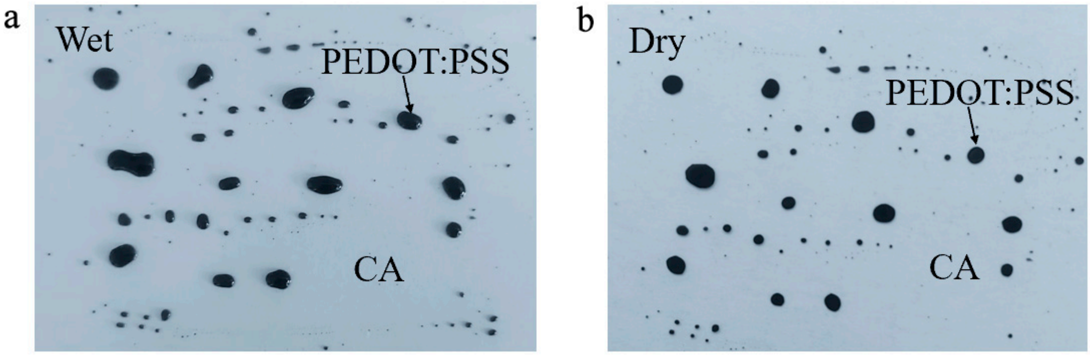

Figure S1. Photos of (a) wet PEDOT:PSS on CA film and (b) dry CA/PEDOT:PSS film.

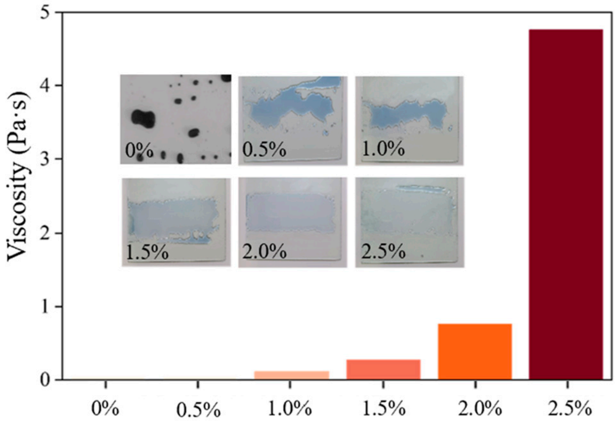

Figure S2. Effect of CMC concentrations on the viscosity of CMC/PEDOT:PSS solution.

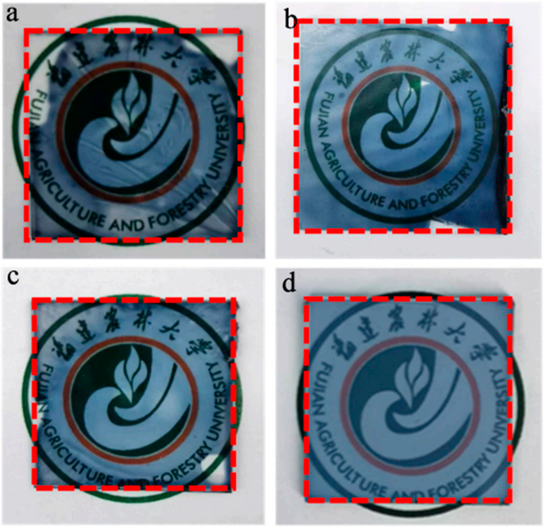

Figure S3. Photo of CPC with various-time coatings: (a) 3rd, (b) 6th, (c) 7th, (d) 9th sample.

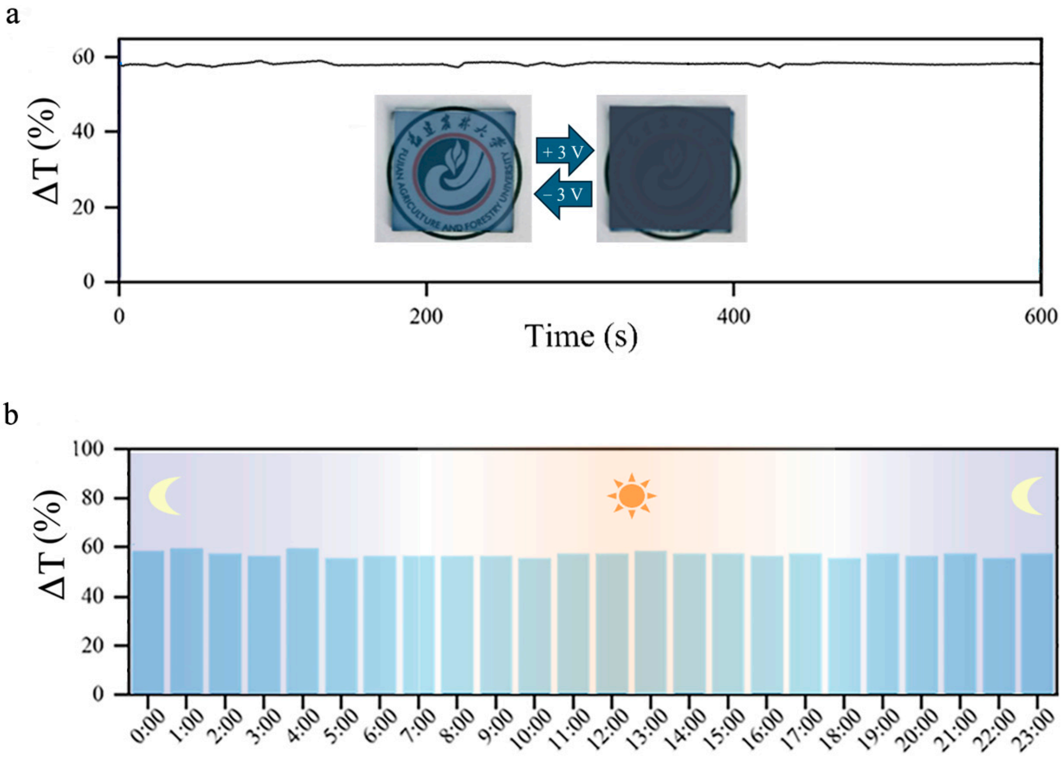

Figure S4. Optical modulation capability of the CPC film (a) under 80-time color–decoloring cycle tests; (b) under 24 h outdoor test.

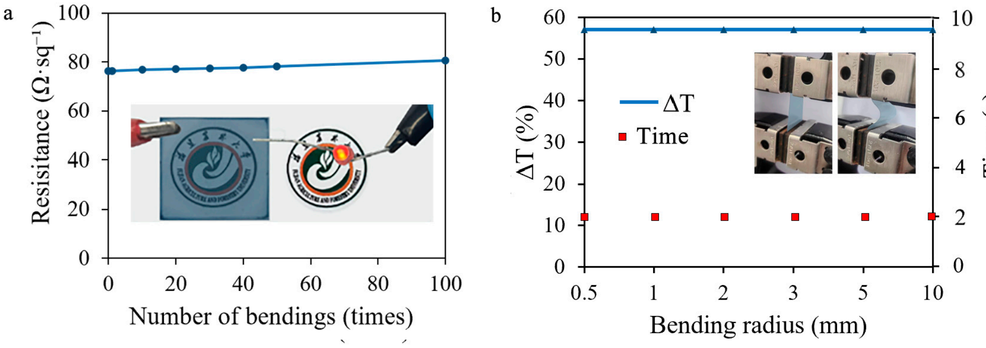

Figure S5. (a) Resistance changes of CPC film after bending cycle tests under a 10 mm bending radius. (b) Optical modulation capability and response time of the CPC film after 100-time bending cycle tests under various bending radii.

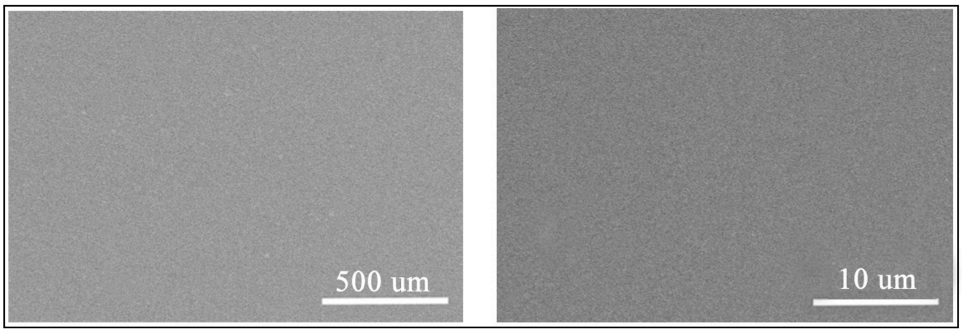

Figure S6. SEM images of the CPC film after 100-time bending cycles under a 10 mm bending radius.

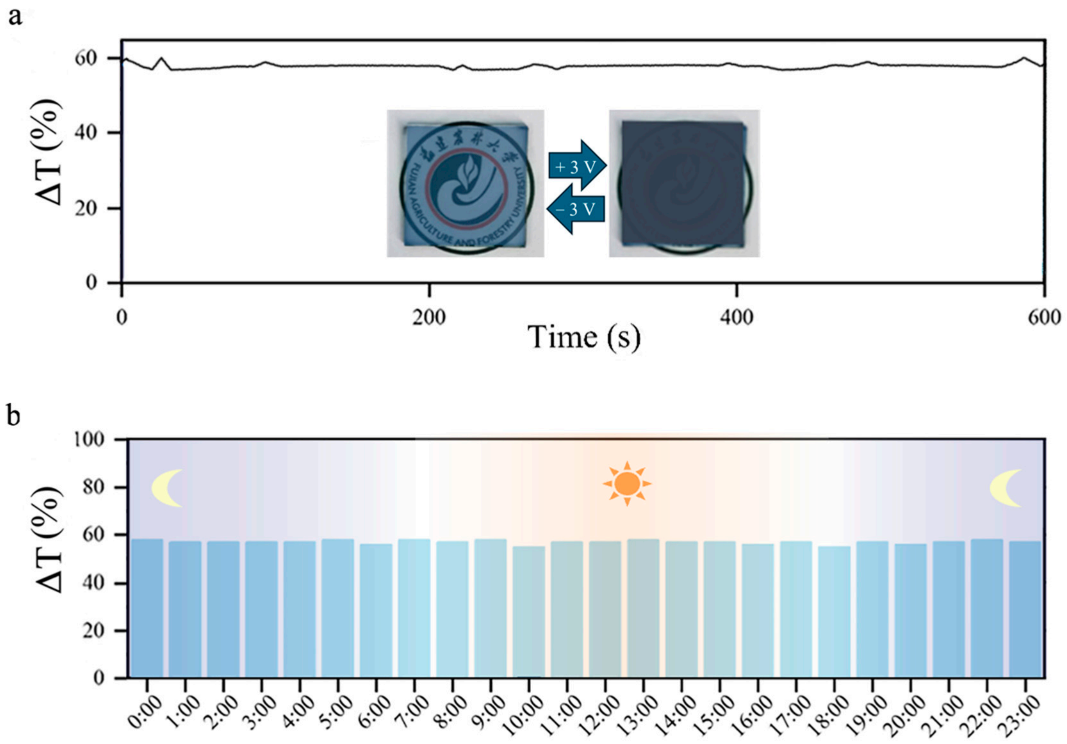

Figure S7. Optical modulation capability of the CPC film after 100-time bending cycles. (a) Under 80-time color–decoloring cycle tests. (b). Under 24 h outdoor test.

Table S1. Variations in temperature and humidity during the 24 h outdoor test.

Time	Temperature (°C)	Humidity (%)
1:00	27.4	90
2:00	27.2	93
3:00	26.7	94
4:00	26.9	91
5:00	27	89
6:00	27.1	89
7:00	29.1	82
8:00	30.5	74
9:00	31.8	72
10:00	32	72
11:00	33.6	64
12:00	34.4	56
13:00	35.2	60
14:00	35	56
15:00	32.5	61
16:00	29.7	73
17:00	29.6	74
18:00	27.3	89
19:00	27.3	92
20:00	26.8	98
21:00	27.1	92
22:00	27.6	89
23:00	27.6	90
24:00	27.3	93
